# Live-cell super-resolution microscopy reveals a primary role for diffusion in polyglutamine-driven aggresome assembly

**DOI:** 10.1074/jbc.RA118.003500

**Published:** 2018-11-06

**Authors:** Meng Lu, Luca Banetta, Laurence J. Young, Edward J. Smith, Gillian P. Bates, Alessio Zaccone, Gabriele S. Kaminski Schierle, Alan Tunnacliffe, Clemens F. Kaminski

**Affiliations:** From the ‡ Cambridge Infinitus Research Center, Department of Chemical Engineering and Biotechnology and; the § Department of Chemical Engineering and Biotechnology, University of Cambridge, Cambridge CB3 0AS, United Kingdom and; the ¶ Sobell Department of Motor Neuroscience and Movement Disorders and Huntington's Disease Center, Institute of Neurology, University College London, London WC1N 3BG, United Kingdom

**Keywords:** protein aggregation, transport, molecular dynamics, molecular imaging, molecular modeling, protein misfolding, aggresome formation, amyloid protein, live cell SIM, passive transport

## Abstract

The mechanisms leading to self-assembly of misfolded proteins into amyloid aggregates have been studied extensively in the test tube under well-controlled conditions. However, to what extent these processes are representative of those in the cellular environment remains unclear. Using super-resolution imaging of live cells, we show here that an amyloidogenic polyglutamine-containing protein first forms small, amorphous aggregate clusters in the cytosol, chiefly by diffusion. Dynamic interactions among these clusters limited their elongation and led to structures with a branched morphology, differing from the predominantly linear fibrils observed *in vitro*. Some of these clusters then assembled via active transport at the microtubule-organizing center and thereby initiated the formation of perinuclear aggresomes. Although it is widely believed that aggresome formation is entirely governed by active transport along microtubules, here we demonstrate, using a combined approach of advanced imaging and mathematical modeling, that diffusion is the principal mechanism driving aggresome expansion. We found that the increasing surface area of the expanding aggresome increases the rate of accretion caused by diffusion of cytosolic aggregates and that this pathway soon dominates aggresome assembly. Our findings lead to a different view of aggresome formation than that proposed previously. We also show that aggresomes mature over time, becoming more compacted as the structure grows. The presence of large perinuclear aggregates profoundly affects the behavior and health of the cell, and our super-resolution imaging results indicate that aggresome formation and development are governed by highly dynamic processes that could be important for the design of potential therapeutic strategies.

## Introduction

The misfolding of polypeptides and their subsequent aggregation into insoluble amyloids is a characteristic of many neurodegenerative diseases (1, 2). Polyglutamine (polyQ)^4^ is generated by the expansion of clustered glutamine codons in various unrelated protein-coding genes and is implicated in neurodegenerative conditions such as Huntington's disease (HD). Accumulation of misfolded polyQ leads to the formation of aggregates in cytosol and aggresomes near the microtubule organizing center (MTOC) (3), but the details of the protein aggregation process remain unclear. In a previous study (4), we showed that cells first generate cytoplasmic aggregates, which coalesce into aggresomes, which take up a considerable volume in the cell and can lead to DNA damage and interference with the cell cycle (5, 6). Mechanistic information on the assembly process of misfolded proteins is clearly of pathological significance, and investigations at the molecular scale are critical to a detailed understanding of this process. Conventionally, the kinetics of amyloid aggregation reactions are studied in the test tube. *In vitro* experiments have for example demonstrated how monomers of polyQ proteins assemble at seeding sites, leading to elongation of fibrillary aggregates (7, 8). Such fibrils grow to 1–2 μm in length *in vitro*, and they feature a well-defined morphology and are subsequently bound into loosely compacted bundles (9, 10).

*In vitro* experiments enable perfectly adjusted solution conditions, where protein nucleation, diffusion, and elongation kinetics can be tightly controlled (11). However, the intracellular environment is far more complex, featuring active transport, multiple phases, molecular crowding, and compartmentalization, all of which likely affect the kinetics and attributes of protein aggregate formation (12). Therefore, although *in vitro* assays are a convenient tool, their relevance to the physiological situation needs to be examined. In cells, polyQ aggregates appear to be structurally heterogeneous, being composed of a mixture of granules, straight and tortuous filaments, and fibrils (13). Intriguingly, fibrillar structures in cells are typically 7–8 nm in diameter, similar to their *in vitro* counterparts, but their length rarely exceeds 300 nm or so (14). They are thus morphologically similar to those formed *in vitro* but of significantly reduced length (10). In terms of dynamics, intracellular aggregates display distinct patterns that differ fundamentally from their in-solution counterparts. A previous study has demonstrated the remarkable mobility of polyQ aggregates within the cell nucleus, and these intranuclear aggregates were shown to disrupt normal patterns of gene expression (15). In the current paper we focus on the formation of aggresomes in the cytosol. We investigate the nucleation and expansion phases of aggresomes in the perinuclear region and distinguish active from passive transport phenomena. Using a combination of advanced optical imaging modalities, including high speed structured illumination microscopy (SIM), single particle tracking (SPT), and mathematical modeling of aggregate transport in the cell, we establish that aggresome formation is initiated by active transport of small aggregates, which are dispersed throughout the cytosol, to the MTOC. However, at later stages aggresome expansion is mainly driven by diffusion of protein aggregates.

## Results

### Aggresomes expand in volume by recruitment of cytosolic polyQ clusters

We have previously established stable HEK cell lines expressing a tetracycline-inducible partial exon 1 sequence of HDQ72 (huntingtin protein with an expanded polyQ region of 72 glutamine residues) fused to the SNAP-tag protein or to enhanced GFP (EGFP) (6). With continuous induction of HDQ72, intracellular polyQ aggregates, including perinuclear aggresomes, begin to appear within a week (Fig. S1*A*), although the proportion of the cell population containing aggregates remains more or less constant at ∼30% (Fig. S1*B*). This suggests that any aggregate-containing cells that die are replaced by newly divided cells, in which aggregation is already underway or is imminent (6).

**Figure 1.**
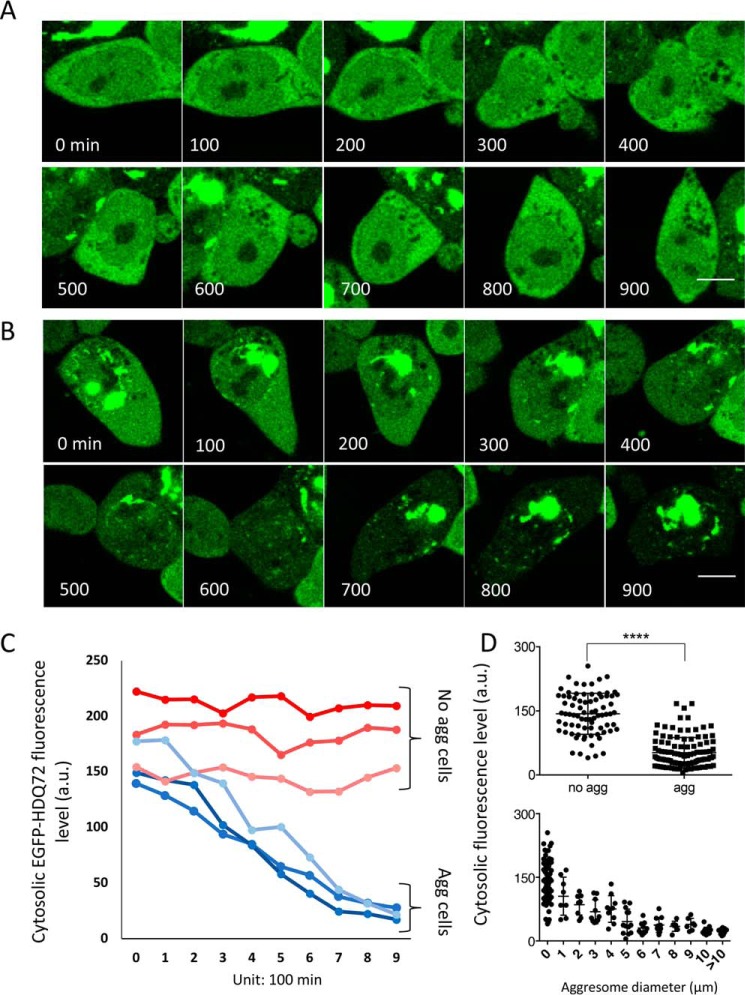
**Perinuclear aggresomes sequester and deplete soluble EGFP-HDQ72 from the cytosol.** *A*, time-lapse images of a cell in which no aggregation takes place over a period of 15 h (900 mins). There is no noticeable decrease of the cytosolic fluorescence signal, indicating that the concentration of EGFP-HDQ72 remains constant. *Scale bar*, 10 μm. *B*, time-lapse images recorded by confocal microscopy of an aggregate-containing cell over a time period of 15 h. The cytoplasmic concentration of soluble EGFP-HDQ72 protein is seen to decrease over time, as it is subsumed into the large perinuclear aggresome. *Scale bar*, 10 μm. *C*, plot of the fluorescence intensity change for recordings such as shown in *A* and *B*. Results are shown for three cell samples containing aggresomes (*blue colors*) and three that were devoid of aggresomes (*red colors*). *D*, *upper panel*, average fluorescence intensity from the cytosol of aggresome-free (*n* = 78) and aggresome-containing (*n* = 106) cells. The *error bars* correspond to standard deviations from the mean. **** indicates a *p* value of <0.0001 in an unpaired *t* test. *Lower panel*, correlation of average cytoplasmic fluorescence intensity with the size of perinuclear aggresomes observed (*n* = 106).

To assess aggregate formation in real time, we performed time-lapse imaging of cells expressing EGFP-HDQ72, with image capture every 15 min over a 15-h period (900 min) (Fig. 1, *A* and *B*). We observed the appearance and gradual increase in size of a compact perinuclear aggresome (Fig. 1*B*), while the fluorescence of the rest of the cytoplasm simultaneously decreased (Fig. 1, *B* and *C*). In contrast, the fluorescence intensity in cells without aggregates remained constant throughout this time period (Fig. 1, *A* and *C*). Quantification of cytoplasmic fluorescence intensity, excluding the main aggregation site, revealed lower fluorescence intensity in aggregate-containing cells compared with nonaggregate containing cells (Fig. 1*D*, *upper panel*). Furthermore, plotting the average cytoplasmic fluorescence against the size of aggregates showed these parameters to be inversely correlated with each other (Fig. 1*D*, *lower panel*). Taken together, these findings suggest that large polyQ aggresomes are formed in the perinuclear region, presumably by continual accretion of monomers or small aggregates from the cytosol. In addition, even when polyQ protein is continuously expressed, a large aggresome seems able to clear the cytoplasm of the majority of smaller aggregates.

### Aggregate assembly in cells is determined by an interplay of diffusion and active transport processes

**Figure 2.**
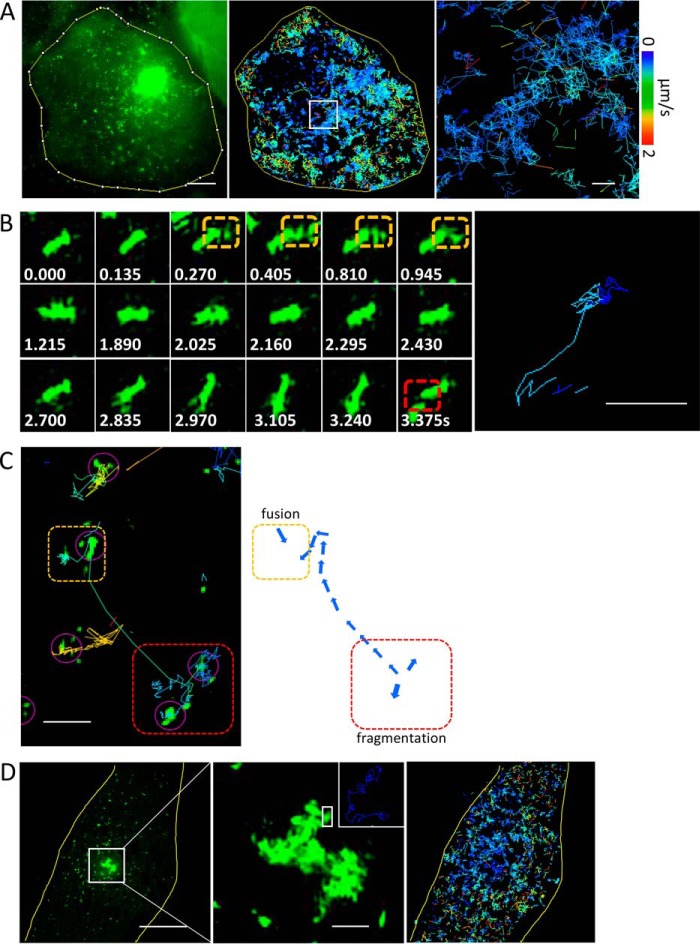
**PolyQ aggregates undergo frequent fusion and fragmentation events in the cell.** *A*, high-speed SIM recordings of intracellular aggregate dynamics. All small aggregates are subjected to dramatic random movements, which lead to collisions and contacts among clusters. Using an SPT algorithm, we identified individual particles and analyzed their trajectories in time at a frame rate of 5 Hz (*middle panel*). The *right-hand panel* shows a zoomed-in version of the region in the *white rectangle*. The velocity spectrum shown applies to all panels. *Scale bars*, 5 μm (*left panel*) and 1 μm (*right panel*). *B*, example images of individual particles in quasirandom motion (6 Hz). The image on the *right* demonstrates the aggregate trajectories. *Numbers* in *white* show the time (in seconds) of each frame. *Dashed orange* and *red rectangles* highlight fusion and fragmentation events, respectively. *Scale bars*, 500 nm. *C*, aggregate motion includes both active and passive transport components. The *purple arrows* highlight regions where small clusters where identified in the automated analysis. The *dashed orange* and *red rectangles* indicate regions where fusion and fragmentation take place, respectively. *Scale bar*, 2 μm. *D*, a cell containing an aggresome in the center and small aggregates in cytosol (*left panel*). A zoomed-in region shows that the loose clusters in the periphery of the aggresome are highly mobile (*middle panel*). The *inset* in the *top right* of the *middle panel* shows motion paths of individual fragments within the aggresome, which are highlighted in the *white square*. SPT reveals that the mobility of aggregate fragments is higher in the in the cytosol than in the aggresome (*right panel*). *Scale bars*, 5 μm (*left panel*) and 1 μm (*middle panel*).

The growth of perinuclear aggresomes at the expense of the cytosolic polyQ fraction led us to investigate how monomeric HDQ72 or small aggregates are added to the perinuclear site. To address this, we performed high-resolution spatiotemporal imaging of aggregation events by SIM (16) using a custom-built setup, capable of 90-nm spatial resolution at frame rates of up to 22 Hz (17). The resulting time-lapse videos revealed that cytosolic polyQ aggregates are small compact structures that are clusters of short fibrils and highly branched and labile in nature, frequently undergoing rapid motion (Video S1), with a size that rarely exceeds ∼500 nm in scale (Fig. 2). Therefore, we define these small aggregated species as aggregate clusters. Using a SPT algorithm, we identified individual aggregate clusters and analyzed their trajectories over a 24-s period at a frame rate of 5 Hz (Fig. 2*A*). The data revealed motion that is overwhelmingly random in fashion for most of the clusters identified. Individual particles moved at speeds ranging from 0 to 2 μm s^−1^ and were initially in localized domains where they fused with other clusters, as shown in Fig. 2*B* and Video S2. In addition to random movement, a small proportion of aggregates appeared to be transported actively, as indicated by rapid linear movements over distances up to 8 μm. In total, less than 3% of all aggregates were found to undergo active transport, which is characterized by linear and long distance (≥2 μm) motion and is totally inhibited by nocodazole (10 μm for 1 h; Fig. S2). Fig. 2*C* shows both passive (diffusional) and active transport events for small clusters. The zoomed regions show that passive transport can lead to both the fragmentation of aggregate clusters (*red rectangle*) and their fusion (*orange rectangle*). The former is suggestive of shear forces prevailing in the dense and multiphasic cellular environment. Unlike the situation *in vitro*, the size of the elongated aggregates within cells appears to be limited by fragmentation: only 1% of clusters were observed to extend beyond 1.5 μm in size, and 90% of all clusters were less than 500 nm in size (Fig. S3). The fusion revealed that small aggregates cluster by fusion of different aggregate particles (*orange rectangles* in Fig. 2, *B* and *C*). Even within large aggresomes, individual small clusters were in motion (Fig. 2*D* and Video S3), although at slower speeds than their freely diffusing counterparts (Fig. 2, *B* and *C*). There is also evidence of active transport in Fig. 2*C*: along the *green track* shown in the *left panel*, one observes highly directed motion, likely along a microtubule (MT).

It was previously suggested that small aggregates are transported along MTs to form aggresomes at the MTOC, because MT inhibition via nocodazole treatment resulted in a pronounced reduction in the formation of aggresomes (3, 18, 19). Our data provide strong and direct evidence of aggregate transport along directional tracks, likely along MTs. We measured active transport at velocities averaging at ∼1 μm s^−1^, which is of the order expected for microtubule-assisted transport by motor proteins (20, 21). Although active transport represents only a very small proportion of cluster movements, over long periods of time (>16 h) most cytosolic clusters had translocated to a perinuclear region near the MTOC. The most parsimonious explanation of this observation is that diffusion is responsible for this deposition of aggregated polyQ protein at the aggresome. Indeed, as shown below, this is likely to be the predominant mechanism, because, as shown in Fig. 1*B*, almost all of the cytosolic polyQ is adsorbed onto the aggresomes after 16 h. In summary, our recordings reveal highly dynamic interplay between fragmentation and fusion and between diffusional and active transport, all of which affect the formation and fate of small aggregate clusters within cells.

### A small proportion of aggregate clusters bind to HDAC6 and MTs

**Figure 3.**
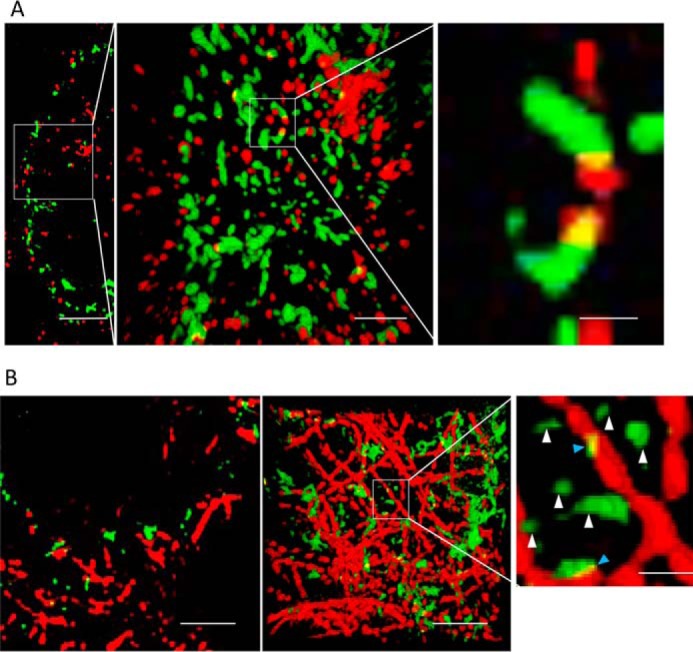
**The majority of polyQ aggregates do not colocalize with HDAC6 or microtubules.** *A*, *left panel*, a section slice showing cytosolic aggregates (*green*) in HEK cells that are immunostained against HDAC6 (*red*). *Middle panel*, projected view from a 3-D rendering of the data corresponding to the marked region in the *left panel. Right panel*, magnified view of polyQ aggregate clusters contained in the region indicated in the *middle panel* to which HDAC6 is bound. Only a small fraction of the clusters colocalize with HDAC6. *Scale bars* from *left* to *right*, 4, 1, and 300 nm, respectively. *B*, *left panel*, a section slice of cytosolic aggregates (*green*) in HEK cells that are immunostained against α-tubulin (*red*). *Middle panel*, same field of view, projecting full 3-D data set. *Right panel*, magnified view of small polyQ clusters localized near microtubules (*blue arrowheads*). The *yellow color* indicates areas where particles are in close association. *White arrows* point to “free” aggregates not associated to microtubules (*white arrowheads*). *Scale bars* from *left* to *right*, 2, 2, and 400 nm, respectively.

Previous studies reported that cytosolic aggregates are recruited by HDAC6 and bind to dynein for active transport along MTs (22, 23). Therefore, to gain further information on the degree of active transport of polyQ clusters in our model, we performed immunostaining against HDAC6 and tubulins in HDQ72-expressing cells, followed by super-resolution imaging and 3-D reconstruction. In Fig. 3*A*, 3-D rendering of the zoomed-in region shows dense distributions of aggregate clusters (*green*) and HDAC6 (*red*); however, aggregate clusters recruited by HDAC6, which display overlapped regions in *yellow* (Fig. 3*A*, *right panel*), are not widely observed. We quantified the proportion of clusters recruited by HDAC6 and found that they represent ∼11% of all aggregates. In Fig. 3*B*, super-resolved structures of MTs (*red*) and aggregate clusters (*green*) demonstrate similar results, showing that ∼9% of aggregates are attached to tubulins (highlighted by *blue arrows* in the *right panel*). These numbers are higher than the fraction of aggregate clusters that were observed to undergo directional transport (3%, see earlier section). The difference may suggest that some MT-associated transport appears random in motion as reported in Ref. 24) and may thus not have been discriminated in the motion analysis of the SPT tracks.

### Large perinuclear aggresomes comprise adsorbed amorphous polyQ clusters that arrive from the cytosol

**Figure 4.**
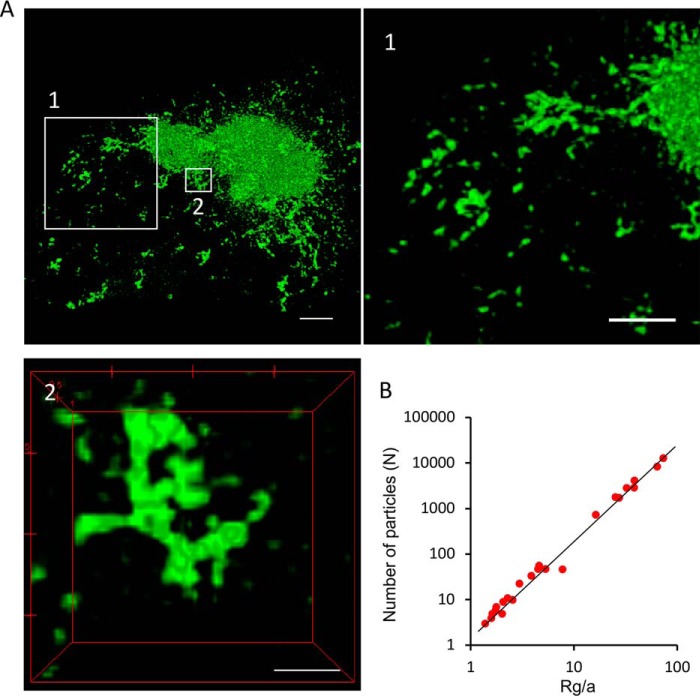
**High-resolution optical imaging of the morphology of an intracellular HDQ72-SNAP aggresome.** *A*, high-resolution SIM images of an aggresome and its peripheral regions. The highlighted regions (*boxes 1* and *2*) are shown in the corresponding panels. *Scale bars*, 2 μm. *Panel 1*, zoomed-in view of peripheral region of the aggresome containing individual, unconnected aggregate fragments that are not associated with the main body of the aggresome. *Panel 2*, example of amorphous small protrusions from the aggresome surface, containing fused aggregate fragments that have arrived from the cytosol. *Scale bars*, 1 μm (1) and 500 nm (2). *B*, number of particles *N* in an aggregate as a function of cluster radius of gyration *R*_g_ for all aggregate clusters, from which the fractal dimension *D*_f_ is inferred; *R*_g_/*a* is the gyration radius normalized to the radius of cluster unit. *a* refers to the radius of an elementary building block made up of small aggregate clusters that, for modeling purposes, is assumed to be spherical.

Super-resolution microscopy offers morphological and structural details of large perinuclear aggresomes. Fig. 4 shows a perinuclear aggresome, imaged with 3-D SIM (Fig. 4*A* and Video S4), which reveals amorphous structures near the aggresome core, with small, isolated clusters appearing in the periphery (Fig. 4*A*, zoomed in regions in *boxes 1* and *2* and Video S5). The small polyQ aggregate clusters feature a very different morphology from those observed in the test tube, *e.g.* in fibril elongation assays (25, 26), or the fibril bundles formed in solution (27), both of which feature clearly defined linear structures. Instead, the cytosolic clusters we observe here have multiple, tangled branches and are limited in size to less than 1.5 μm. The appearance of a dense core region of the aggresome and a less compacted peripheral region containing discernible individual species led us to speculate that individual small clusters are recruited to the large perinuclear aggresomes located at the MTOC (3) and that this association and the ensuing aggresome growth and compaction are responsible for the consumption of cytoplasmic protein material presented in Fig. 1.

Aggresomes are assembled by small clusters in an irreversible and partially disordered fashion by diffusion, which leads to their amorphous and tangled morphology. Assembly of amorphous aggregates in this fashion has been shown to lead to structures with fractal morphologies (28). To test whether this premise is true in the present case and to give further credence to the hypothesis that aggresome development is primarily governed by diffusion driven phenomena, we performed a fractal analysis on the evolving aggresome topologies. We calculated the fractal dimension *D*_f_, relating the cluster mass to the evolving cluster sizes (see “Experimental procedures: and Fig. S4). The results show that the radius of gyration (used as a characteristic measure of cluster size) scales linearly with the number of small clusters identified within them, an observation typical of fractal objects (Fig. 4*B*). In what follows, the fractal dimension is used in a computational model of aggresome formation.

### Mathematical modeling of aggresome formation and expansion

To gain a better understanding of our experimental observations, we developed a mathematical model describing the formation and growth of perinuclear aggresomes, which included both diffusion and active transport processes. We considered an aggresome to be a spherical fractal ensemble of elementary building blocks, resembling the experimental observation of cytosolic clusters assembling into a fractal superstructure. Transport was modeled using a diffusion equation to mimic Brownian motion of aggregate clusters in the cytosol and a pure advection equation for active transport to the MTOC along MTs. The model reduces to a moving-boundary transport problem where two distinct motion processes take place at the same time. This leads to two Cauchy problems, linked by overall matter conservation, for diffusion and active transport, respectively, in a spherical geometry. Conservation of matter at the solid–liquid (or aggresome surface) interface is implemented to describe the quantity of polyQ clusters migrating into the growing aggresome and to update the position of the radius of the expanding aggresome (the moving lower boundary of the domain where the differential equations are solved). Full details of the computational approach are presented in the supporting Information.

**Figure 5.**
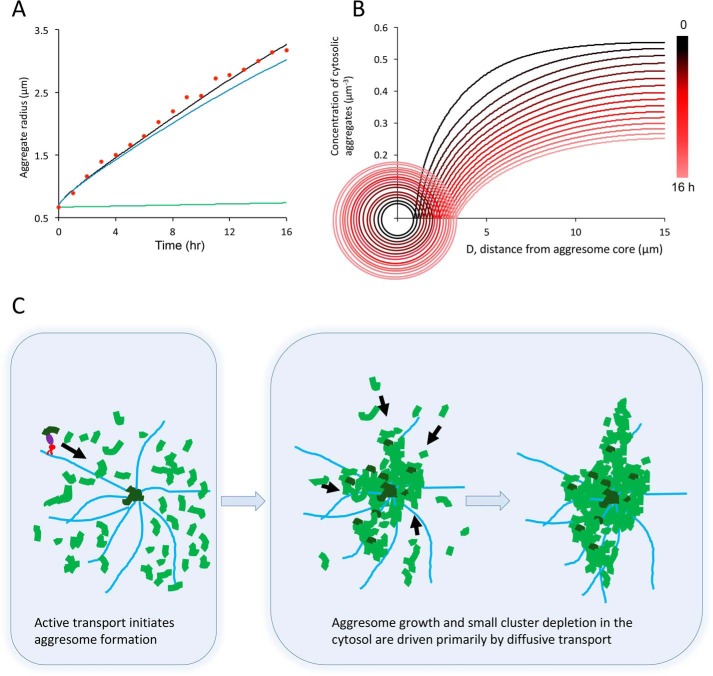
**Mathematical modeling of aggresome formation.** *A*, results from a theoretical model of aggresome expansion over time with contributions from active transport (*green solid line*) and diffusion (*blue line*). The composite model (*black line*) provides a good fit to the experimental data points (*red dots*). The model confirms that the growth trend is dominated by the contribution of passive transport to the aggresome. *B*, model results indicating the concentration of cytosolic aggregate clusters as a function of time (from 0 to 16 h, corresponding colors changing from *black* to *red*). The *circles* indicate the size of the expanding aggresome at different time points. At the aggresome surface, the cytosolic concentration of clusters drops to zero. At large distances the cytosolic concentration of aggregate material is seen to decrease in time, as material is consumed by the growing aggresome. *C*, proposed model for aggresome formation, summarizing evidence from experimental and model observations. The expression of aggregation-prone HDQ72 protein leads to the formation of accumulation of aggregate clusters in the cytosol. Clusters recruited by HDAC6 (*purple*) undergo dynein (*red*) and MT-dependent active transport (*black arrow*). Aggregate clusters actively transported to the MTOC form a nucleation core on which other aggregate clusters can be adsorbed. As the aggresome expands in size, its increasing surface area increases the probability for capture of diffusing aggregates, and eventually diffusion becomes the dominant process by which aggregating material arrives to drive aggresome expansion. Aggresome expansion driven by diffusion is irreversible, leading to the eventual depletion of cytosolic HDQ72 as observed in experiments. MTs are shown in *light blue*, cytosolic aggregate clusters undergoing diffusion are in *light green*, and aggregate clusters undergoing active transport are in *dark green*.

Fig. 5*A* compares the experimentally measured growth of aggresomes over a 16-h span with the model results. The full model (*black line*) comprises both active transport (*green line*) and diffusion (*blue line*) components and provides an excellent fit to the experimental data (*red dots*). The model clearly demonstrates that aggresome growth is dominated by diffusion, although inclusion of active transport is required in a comprehensive model of the process. Next, we correlated the aggresome expansion in time with the concentration of aggregate clusters in the cytosol (Fig. 5*B*). As time increases (color of curves changing from *black* to *red*), the cytosolic concentration is clearly seen to decrease (decrease in plateau region of curves, at far distance). For each time point, the cytosolic concentration is also seen to decrease with proximity to the aggresome surface; thus the process exhibits clear source-sink behavior. The trends captured in the model shown in Fig. 5*B* are consistent with the results from the time-lapse imaging shown in Fig. 1*B*, which also shows a continuously decreasing cytosolic of polyQ concentration as aggresome grow in size. Recruitment of clusters leads to aggresome expansion and depletion of cytosolic clusters. It is interesting to note that in some cells, the cytosolic polyQ concentration was not observed to change in time, and no substantial aggresomes formed (Fig. 1*B*). This observation, in conjunction with the modeling results, suggests that there exists a delicate balance of cluster formation and removal rates in the cell, which will be discussed in further detail in the following sections.

### The aggresome core becomes increasingly dense over time

**Figure 6.**
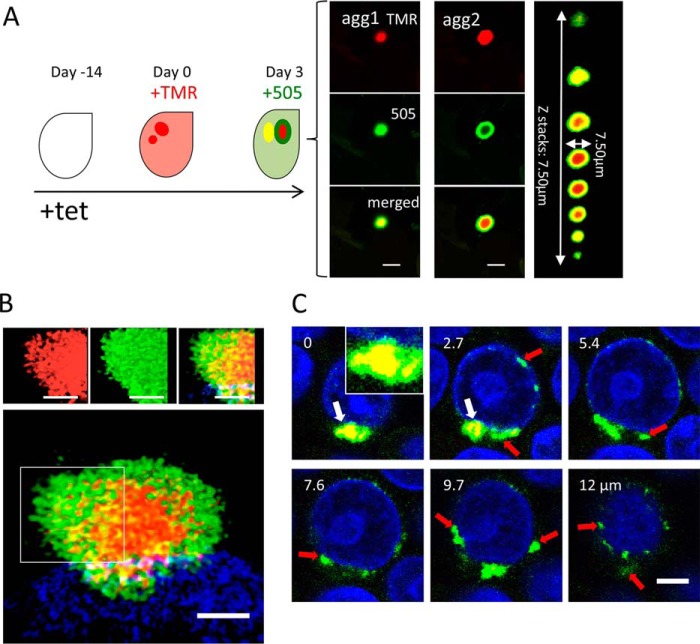
**Dynamic imaging of aggresome growth.** *A*, cartoon to demonstrate the two-color labeling protocol. Cells expressing SNAP-HDQ72 were labeled with TMR-Star at day 0 (*red color*) and 3 days later with 505-Star (*green*). Confocal images reveal two types of aggresome. In early-stage aggresomes, newly added material diffuses into core domains, leading to complete overlap of the two colors in the images. In contrast, late-stage aggresomes become increasingly impermeable (dense), and the newly adsorbed material remains spatially distinct from the core region. The merged *Z*-stack was obtained by cropping over a region containing the aggresome from the original confocal image stack. This region was then rendered by the 3-D viewer in Fiji, and the contrast was adjusted to highlight the extent of the aggresome. *Scale bar*, 5 μm. *Right panel*, confocal slices for agg2. *B*, 3-D SIM images of an impermeable aggresome. Labeling protocol as in *A*. Both the inner part and peripheral regions of aggregates were composed of small clusters. *Scale bar*, 2 μm. The cells were stained with Hoechst 33342 (*blue*). *C*, sequential labeling with alternating dyes reveals that the core region becomes increasingly impermeable over time. *Scale bar*, 7.5 μm. The cells were stained with Hoechst 33342 (*blue*).

To further validate our observation and modeling that perinuclear aggresomes grow by accretion of cytoplasmic material, we devised an experimental system to visualize the addition of newly synthesized polyQ protein onto existing perinuclear aggresomes using a stable cell line for inducible expression of SNAP-tagged HDQ72 protein (5). In this system, newly synthesized protein of interest can be irreversibly, covalently, and sequentially labeled by two different dyes (29). Thus, we induced the expression of SNAP-HDQ72 with tetracycline for 14 days to allow aggregates to form and then added TMR-Star (580-nm emission peak, *red*) to the culture for 30 min to label the HDQ72 protein already synthesized within the cells. We then replaced the medium containing TMR-Star and grew the cells for 3 more days without label before adding 505-Star (532-nm emission, *green*) for 30 min. This allowed newly expressed HDQ72 protein to be labeled with a different color. After washing out 505-Star, the cells were incubated at 37 °C for another 2 h to stabilize them before fixation with ice-cold methanol followed by confocal microscopy (Fig. 6*A*).

We identified two types of aggresome in this way. First, aggresomes were observed in which the core was labeled by both colors, suggesting that the newly expressed HDQ72 could enter the core region of an already formed aggresome (agg1 in Fig. 6*A*). Second, we observed aggresomes with a *red core* surrounded by a *green shell* of newly added HDQ72, indicating that such aggresomes have an impermeable core (agg2 in Fig. 6*A*). A typical aggresome of this type is also shown in a series of confocal *Z*-stack images to provide a 3-D overview (Fig. 6*A*, *far-right panel*). High-resolution SIM imaging (Fig. 6*B*) revealed some mixing of SNAP labels at the interface between core (*red*) and peripheral shell (*green*), consistent with the surface of such aggresomes being less compact than the core, permitting diffusive mixing and intercalation of fragments on the aggresome surface.

We were able to demonstrate, using the labeling scheme in Fig. 6*A* and a series of confocal *Z*-stack images, that although large aggresomes consist of proteins expressed during both labeling periods (Fig. 6*C*, *white arrows*), small aggregates (Fig. 6*C*, *red arrows*) separate from this aggresome were composed only of newly expressed proteins (*green* only). This indicates that small aggregate clusters are either transported to aggresomes or that they are degraded within a few days of synthesis. This directly confirmed our modeling that aggresomes initiate from a nucleation site and then expand rapidly by attracting small aggregate clusters. We also found that aggresomes with an impermeable core comprise less than 20% of the total population after 3 months of gene induction. Compared with permeable aggresomes that vary in size from 1 to 9 μm in diameter, impermeable aggresomes are all >4 μm in diameter, indicating that an impermeable core structure is associated with larger aggresomes. In summary, our two-color labeling experiments suggest that aggresomes mature from permeable, loose structures into structures with more compacted cores. We take two potential conclusions from the observations presented so far. First, aggregated protein in late-stage aggresomes with compacted cores is less likely to dissociate. Second, the increasing surface area of late aggresomes increases the probability that new material will adsorb. Taken together, these phenomena may explain the near-complete depletion of cytosolic polyQ in cells where large aggresomes are present (Fig. 1*B*). It may also explain why in some cells large aggresomes never form, because in these cases fragmentation is dominant over accumulation. These processes are distinctly different from what is observed in test tube experiments.

### The morphology of polyQ aggregates in cell models mimics that of aggregates in a mouse model of Huntington's disease

**Figure 7.**
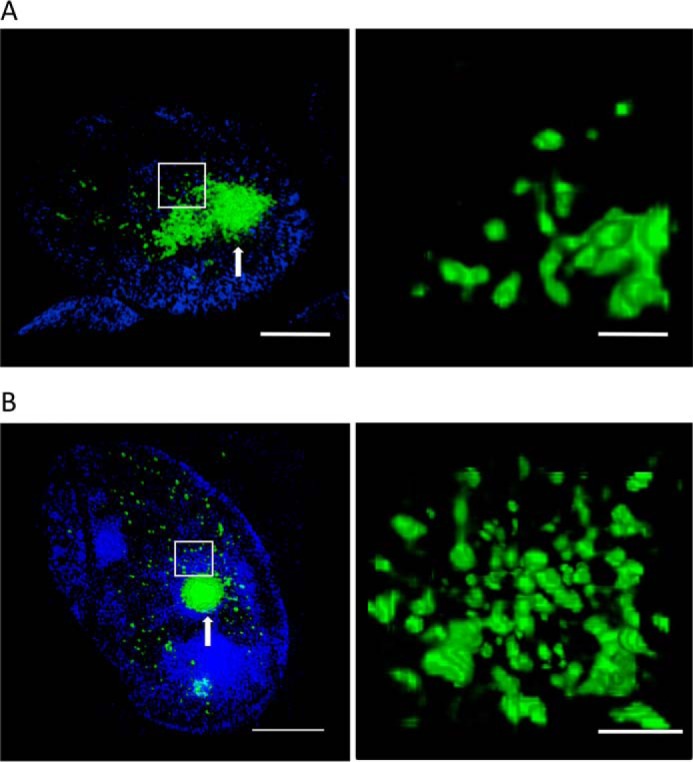
**Intranuclear aggregates in HEK cells have a similar morphology to those observed in an R6/2 mouse model.** *A*, 3-D SIM images of intranuclear EGFP-HDQ72 aggregates (*green*) in the HEK cell model expressing SNAP-HDQ72. Numerous small clusters and a large aggregate were embedded inside the nucleus. The *right-hand panel* shows a zoomed-in version of the region in the *white rectangle. Scale bars*, 2 μm (*left panel*) and 500 nm (*right panel*). The cells were stained with Hoechst 33342 (*blue*). *B*, 3-D SIM images of immunostained intranuclear polyQ aggregates (*green*) in the R6/2 mouse model at 14 weeks of age. The aggregates feature a similar morphology to that observed in the cell model. *Right panel*, zoomed-in region shows the amorphous clusters. *Scale bars*, 2 μm (*left panel*) and 500 nm (*right panel*). The cells were stained with Hoechst 33342 (*blue*).

One of the characteristics of HD is the accumulation of polyQ aggregates in both nuclear and cytoplasmic regions of brain cells. To investigate to what extent our data reflect the morphological details of polyQ structures found in models of HD pathology, we performed experiments in brain sections of the R6/2 mouse, a transgenic strain that expresses a version of exon 1 of the human huntingtin gene that encodes at least 150 glutamine repeats (30). Fig. 7*A* shows a large nuclear aggregate visualized with 3-D SIM using the EGFP-HDQ72 HEK cell model. All medium spiny neurons within the striatum of a 14-week R6/2 mouse have nuclear aggregates that are morphologically homogeneous. The structure is similar in appearance to that of perinuclear aggresomes in that cell model (Fig. 4), although the mechanisms of formation are likely different in the nucleus. The percentage of neurons with nuclear aggregates will depend on the type of neuron and the brain regions examined. The brain sections from the R6/2 mouse were immunostained with the S830 anti-huntingtin antibody and imaged for comparison. In contrast to the HEK cell model, perinuclear aggresomes were less abundant, and large aggregates featured predominantly in the nuclear domain. However, the morphology of nuclear aggregates appeared very similar to that in the HEK cell model (Fig. 7*B*). Again, the aggregates appear highly condensed at their core (*white arrows* in Fig. 7, *A* and *B*) but more loosely structured at their periphery. The experiments confirmed that the morphological diversity of aggregate structures formed in the mouse model is similar to that in the HEK cell model, confirming that the cell line is representative of protein aggregation in models of HD, thus providing a convenient means to study molecular aspects of the disease and therapeutic strategies.

## Discussion

PolyQ fibril formation kinetics have been widely studied both *in vitro* under controlled solution conditions. However, polyQ aggregates formed in cells show different structural characteristics to their *in vitro* counterparts. For example, elongation into long fibrillary structures has not been observed in the cell, and fibrils do not usually extend significantly beyond a mean length of ∼125 nm (14), in stark contrast to their solution counterparts, which can extend to several microns in length (9, 10). Our experiments demonstrate that the assembly of short fibrils into larger structures is primarily driven by diffusion rather than elongation by end-on monomer addition but also involved fast fusion and fragmentation events that lead to the formation of branched clusters of ∼500–1000 nm in diameter.

This, together with other data we report here, leads to a different view of aggresome formation than proposed previously. It has been suggested that aggresome formation depends on HDAC6- and dynein-facilitated active transport along MTs (22). Although we find that this is an important first step in the nucleation of aggresomes, subsequent aggresome expansion is primarily driven by diffusion. A model of aggresome formation shows that initially, rates of aggregate accumulation via active transport and diffusion are comparable (Fig. 5*A*), but they rapidly diverge once nucleation has occurred (time 0 in Fig. 5*A*). The nascent aggresome near the MTOC grows in size and forms amorphous and fractal objects (Fig. 4*B*) with rapidly increasing surface area (Figs. 1*B* and 5*B*). This accelerates the capture rate for new aggregate material arriving by diffusion (Fig. 5*B*), and this gradually becomes the primary route for aggresome expansion (Fig. 5, *A* and *C*). The model explains why in some cells the soluble and cytosolic fraction of polyQ gets rapidly depleted, because the rate of consumption by the aggresome exceeds the production of new protein (see Figs. 1*B* and 5*B* for experimental and model data, respectively). On the other hand, some cells do not form aggresomes at all, and the initial nucleation near the MTOC never takes place. This suggests that in these cells degradation mechanisms, *e.g.* by lysosomal or ubiquitin-proteasome system (UPS)–mediated degradation of monomeric species, are sufficiently strong to prevent the formation of cytosolic aggregate clusters, and this prevents their subsequent assembly into aggresomes. On the other hand, our study shows that transport of small fibrils from the cytosol leads to the nucleation of aggresomes near the MTOC. Strategies for therapeutic intervention should thus focus on preventing the nucleation of small aggregate species in the cytosol, keeping the protein pool soluble in order for UPS degradation to function optimally and thus preventing fibrillary species from arriving at the MTOC. Fibrillar structures are known to be difficult to degrade in the cell (31, 32), and thus their transport to the MTOC just leads to irreversible clustering. This suggests that the UPS that can target monomers for degradation (32, 33, 34) is a key target for the prevention of aggresome formation. The conclusions are consistent with a previous finding that inhibition of the active transport pathway via nocodazole treatment of cells, known to lead to microtubule depolymerization, did indeed prevent aggresome formation near the MTOC; however, it led to protein accumulation and aggregation in the cytosol (3, 18, 19).

In the literature, there is an ongoing debate on the relative toxicities of monomeric and aggregated protein species in the cell (35). Much of the recent literature has focused on soluble species as the main problem in amyloid-mediated neuropathology and suggestions have been made to drive the amyloidogenic protein species into an aggregated and thus more passive state (36, 37). However, the current work suggests that this may not be the right strategy after all: large intracellular aggregates have been shown to produce long term toxicity, for example, by inducing DNA damage and apoptosis (6). Therefore, increasing the rate of fibril formation in the cytosol may be a risky strategy, because this enhances the likelihood of aggresome formation as we show here.

In conclusion, we have shown through a combined approach of advanced experimental analysis and modeling that both ATP-mediated and purely passive, physical phenomena play very important roles in the homeostasis of misfolded protein. The study highlights differences as well as similarities of the protein aggregation processes occurring *in vitro* and in the cell.

## Experimental procedures

### Materials

l-Glutamine, Zeocin, hygromycin, blasticidin, Dulbecco's modified Eagle's medium, PBS, fetal bovine serum, Hoechst 33342 solution, nocodazole (M-1404), and agarose were purchased from Sigma–Aldrich. TMR-Star and 505-Star staining reagents were purchased from New England Biolabs. Antibodies against HDAC6 (7558S) (dilution for immunofluorescence 1:300) were from Cell Signaling Technology, and those against α-tubulin (T5168) (dilution for immunofluorescence 1:300) were from Sigma–Aldrich.

### Cells

Details on plasmids and construction of the stable cell line were as described previously (6). Briefly, mammalian Flp-In T-REx293 cells were grown in T75 or T25 flasks or 6-well plates by incubation at 37 °C in a 5% CO_2_ atmosphere. Complete medium for normal cell growth consisted of 90% Dulbecco's modified Eagle's medium, 10% fetal bovine serum with 2 mm l-glutamine; antibiotics were used as appropriate. The cells were kept in logarithmic phase growth and passaged on reaching 80–90% confluence (approximately every 3–4 days). Medium was changed every 2 or 3 days. The cells were treated with nocodazole (10 μm) for 1 h before imaging.

### Mice

All experimental procedures performed on mice were conducted under a project license from the Home Office (Animal Scientific Procedures Act 1996) and approved by the King's College London Ethical Review Process Committee. Hemizygous R6/2 mice (30) were bred by backcrossing R6/2 males to (CBA × C57BL/6) F1 females (B6CBAF1/OlaHsd; Harlan Olac). The mice were genotyped, and the CAG repeat was measured as previously described (38). The animals were housed under a 12-h light/12-h dark cycle, with unlimited access to water and food (Special Diet Service, Witham, UK) in a conventional unit. Cages were environmentally enriched with a cardboard tube. Animals were sacrificed through terminal anesthesia with Euthatal (Marial, Harlow, UK) at 14 weeks of age. The brains were removed and fixed in 4% Parafix (Pioneer Research Chemical Ltd., Essex, UK) for 48 h. The brains were then rinsed in PBS and stored in 30% sucrose, 0.05% sodium azide in PBS until sectioning. Coronal sections from a female mouse (CAG = 215) and WT control were taken serially at 50-μm thickness on a freezing microtome (HM430 Microm, Thermo Scientific) and stored at −20 °C in tissue cryoprotective solution containing 0.05% sodium azide until staining.

Sections were washed in PBS prior to nonspecific binding blocking with 1-h incubation in 10% normal serum with 0.3% Triton X-100 in PBS. They were then incubated overnight at 4 °C in primary antibody against S830 (1:2000) and raised against exon 1 huntingtin with 53 glutamines (39), prior to incubation in secondary Alexa-488 fluorescent dye (Molecular Probes) for 2 h at room temperature. After a subsequent 15-min incubation in Hoechst (Invitrogen) sections were washed in PBS and coverslipped with Vectashield (Vector Laboratories, Peterborough, UK).

### Microscopy

After induction for various times, SNAP-HDQ72-expressing cells were incubated for 30 min at 37 °C with 300 mm SNAP-Cell TMR-Star (New England Biolabs) dissolved in complete medium. After labeling, the samples were washed three times with complete medium and incubated for 30 min prior to imaging. Images were recorded with an OMX V3 super-resolution microscope. Confocal microscopy and immunofluorescence protocols were as described previously (6).

Fixed samples were imaged on a confocal microscope (Leica TCS SP5, using the Leica Application Suite (LAS AF)) with a HCX PL APO 40×/1.25–0.75 oil objective lens (Leica) or HCX PL APO 60×/1.40–0.60 oil objective lens (Leica) at room temperature. Fluorochromes used for individual experiments are stated in the figure legends. For the time-lapse fluorescence intensity measurements, the cells were grown in glass-bottomed Petri dishes at 37 °C in a 5% CO_2_ atmosphere. On the day of imaging, the samples were first stabilized in the incubation chamber of the Leica TCS SP5 system with continuous air supply (37 °C and 5% CO_2_). Complete medium was used for live-cell imaging experiments, and half the medium was replaced every 2 days. The time interval between consecutive image captures varied from 5 to 15 min, as appropriate. The entire setup was controlled using the Leica LAS AF software, and ImageJ was used for image processing and analysis. For the aggresome permeability measurements, we recorded confocal image stacks for both labels used, and only those aggregates were classified as permeable aggregates that were homogenously labeled throughout their volume with both colors.

To visualize intracellular aggregate motion, we used our custom-built SIM providing a spatial resolution approaching 90 nm at frame rates reaching 22 Hz (17). The cells were grown in glass-bottomed Petri dishes at 37 °C in a 5% CO_2_ atmosphere. On the day of imaging, Petri dishes were first stabilized in the incubation chamber of the SIM system with continuous air supply (37 °C and 5% CO_2_). Hardware control and image reconstruction were performed with software written in LabView and Matlab (16, 40). For motion analysis and visualization, ImageJ was used.

## Author contributions

M. L., A. T., and C. F. K. conceptualization; M. L., L. J. Y., E. J. S., G. P. B., A. Z., A. T., and C. F. K. resources; M. L., A. T., and C. F. K. data curation; M. L., L. B., and C. F. K. formal analysis; M. L., A. T., and C. F. K. validation; M. L., L. J. Y., A. T., and C. F. K. investigation; M. L., A. T., and C. F. K. visualization; M. L., L. B., L. J. Y., A. Z., G. S. K. S., A. T., and C. F. K. methodology; M. L., A. T., and C. F. K. writing-original draft; M. L., G. S. K. S., A. T., and C. F. K. writing-review and editing; G. P. B., A. Z., G. S. K. S., A. T., and C. F. K. supervision; G. S. K. S., A. T., and C. F. K. funding acquisition; G. S. K. S., A. T., and C. F. K. project administration.

## Supplementary Material

Supporting Information

## Footnotes

4 The abbreviations used are:

polyQ: polyglutamine
HD: Huntington's disease
MTOC: microtubule organizing center
SIM: structured illumination microscopy
SPT: single particle tracking
EGFP: enhanced GFP
MT: microtubule
UPS: ubiquitin-proteasome system.

## References

[1] Ross C. A., and Poirier M. A. (2004) Protein aggregation and neurodegenerative disease. Nat. Med. 10, S10–S17 10.1038/nm1066 15272267

[2] Ciechanover A., and Kwon Y. T. (2015) Degradation of misfolded proteins in neurodegenerative diseases: therapeutic targets and strategies. Exp. Mol. Med. 47, e147 10.1038/emm.2014.117 25766616 PMC4351408

[3] Johnston J. A., Ward C. L., and Kopito R. R. (1998) Aggresomes: a cellular response to misfolded proteins. J. Cell Biol. 143, 1883–1898 10.1083/jcb.143.7.1883 9864362 PMC2175217

[4] Lu M., Williamson N., Boschetti C., Ellis T., Yoshimi T., and Tunnacliffe A. (2015) Expression-level dependent perturbation of cell proteostasis and nuclear morphology by aggregation-prone polyglutamine proteins. Biotechnol. Bioeng. 112, 1883–1892 10.1002/bit.25606 25854808

[5] Giuliano P., De Cristofaro T., Affaitati A., Pizzulo G. M., Feliciello A., Criscuolo C., De Michele G., Filla A., Avvedimento E. V., and Varrone S. (2003) DNA damage induced by polyglutamine-expanded proteins. Hum. Mol. Genet. 12, 2301–2309 10.1093/hmg/ddg242 12915485

[6] Lu M., Boschetti C., and Tunnacliffe A. (2015) Long term aggresome accumulation leads to DNA damage, p53-dependent cell cycle arrest, and steric interference in mitosis. J. Biol. Chem. 290, 27986–28000 10.1074/jbc.M115.676437 26408200 PMC4646037

[7] Walters R. H., Jacobson K. H., Pedersen J. A., and Murphy R. M. (2012) Elongation kinetics of polyglutamine peptide fibrils: a quartz crystal microbalance with dissipation study. J. Mol. Biol. 421, 329–347 10.1016/j.jmb.2012.03.017 22459263 PMC3572748

[8] Huynen C., Willet N., Buell A. K., Duwez A. S., Jerôme C., and Dumoulin M. (2015) Influence of the protein context on the polyglutamine length-dependent elongation of amyloid fibrils. Biochim. Biophys. Acta 1854, 239–248 10.1016/j.bbapap.2014.12.002 25489872

[9] Streets A. M., Sourigues Y., Kopito R. R., Melki R., and Quake S. R. (2013) Simultaneous measurement of amyloid fibril formation by dynamic light scattering and fluorescence reveals complex aggregation kinetics. PLoS One 8, e54541 10.1371/journal.pone.0054541 23349924 PMC3547910

[10] Duim W. C., Jiang Y., Shen K., Frydman J., and Moerner W. E. (2014) Super-resolution fluorescence of huntingtin reveals growth of globular species into short fibers and coexistence of distinct aggregates. ACS Chem. Biol. 9, 2767–2778 10.1021/cb500335w 25330023 PMC4273975

[11] Kaminski C. F., and Kaminski Schierle G. S. (2016) Probing amyloid protein aggregation with optical superresolution methods: from the test tube to models of disease. Neurophotonics 3, 041807 10.1117/1.NPh.3.4.041807 27413767 PMC4925874

[12] Pinotsi D., Michel C. H., Buell A. K., Laine R. F., Mahou P., Dobson C. M., Kaminski C. F., and Kaminski Schierle G. S. (2016) Nanoscopic insights into seeding mechanisms and toxicity of α-synuclein species in neurons. Proc. Natl. Acad. Sci. U.S.A. 113, 3815–3819 10.1073/pnas.1516546113 26993805 PMC4833232

[13] DiFiglia M., Sapp E., Chase K. O., Davies S. W., Bates G. P., Vonsattel J. P., and Aronin N. (1997) Aggregation of huntingtin in neuronal intranuclear inclusions and dystrophic neurites in brain. Science 277, 1990–1993 10.1126/science.277.5334.1990 9302293

[14] Bäuerlein F. J. B., Saha I., Mishra A., Kalemanov M., Martínez-Sánchez A., Klein R., Dudanova I., Hipp M. S., Hartl F. U., Baumeister W., and Fernández-Busnadiego R. (2017) *In situ* architecture and cellular interactions of polyQ inclusions. Cell 171, 179–187.e10 10.1016/j.cell.2017.08.009 28890085

[15] Li L., Liu H., Dong P., Li D., Legant W. R., Grimm J. B., Lavis L. D., Betzig E., Tjian R., and Liu Z. (2016) Real-time imaging of Huntingtin aggregates diverting target search and gene transcription. eLife 5, e17056 10.7554/eLife.17056 27484239 PMC4972539

[16] Strtohl F., and Kaminski C. F. (2016) Frontiers in structured illumination microscopy. Optica 3, 667–677 10.1364/OPTICA.3.000667

[17] Young L. J., Ströhl F., and Kaminski C. F. (2016) A guide to structured illumination TIRF microscopy at high speed with multiple colors. J. Vis. Exp. 111, e53988 10.3791/53988 27285848 PMC4927749

[18] Taylor J. P., Tanaka F., Robitschek J., Sandoval C. M., Taye A., Markovic-Plese S., and Fischbeck K. H. (2003) Aggresomes protect cells by enhancing the degradation of toxic polyglutamine-containing protein. Hum. Mol. Genet. 12, 749–757 10.1093/hmg/ddg074 12651870

[19] Wang H., Ying Z., and Wang G. (2012) Ataxin-3 regulates aggresome formation of copper-zinc superoxide dismutase (SOD1) by editing K63-linked polyubiquitin chains. J. Biol. Chem. 287, 28576–28585 10.1074/jbc.M111.299990 22761419 PMC3436588

[20] Verhey K. J., Kaul N., and Soppina V. (2011) Kinesin assembly and movement in cells. Annu. Rev. Biophys. 40, 267–288 10.1146/annurev-biophys-042910-155310 21332353

[21] Howard J. (1997) Molecular motors: structural adaptations to cellular functions. Nature 389, 561–567 10.1038/39247 9335494

[22] Kawaguchi Y., Kovacs J. J., McLaurin A., Vance J. M., Ito A., and Yao T. P. (2003) The deacetylase HDAC6 regulates aggresome formation and cell viability in response to misfolded protein stress. Cell 115, 727–738 10.1016/S0092-8674(03)00939-5 14675537

[23] Iwata A., Riley B. E., Johnston J. A., and Kopito R. R. (2005) HDAC6 and microtubules are required for autophagic degradation of aggregated Huntingtin. J. Biol. Chem. 280, 40282–40292 10.1074/jbc.M508786200 16192271

[24] Bálint Š., Verdeny Vilanova I. V., SandovalÁlvarez Á., and Lakadamyali M. (2013) Correlative live-cell and superresolution microscopy reveals cargo transport dynamics at microtubule intersections. Proc. Natl. Acad. Sci. U.S.A. 110, 3375–3380 10.1073/pnas.1219206110 23401534 PMC3587250

[25] Buchanan L. E., Carr J. K., Fluitt A. M., Hoganson A. J., Moran S. D., de Pablo J. J., Skinner J. L., and Zanni M. T. (2014) Structural motif of polyglutamine amyloid fibrils discerned with mixed-isotope infrared spectroscopy. Proc. Natl. Acad. Sci. U.S.A. 111, 5796–5801 10.1073/pnas.1401587111 24550484 PMC4000827

[26] Pinotsi D., Buell A. K., Galvagnion C., Dobson C. M., Kaminski Schierle G. S., and Kaminski C. F. (2014) Direct observation of heterogeneous amyloid fibril growth kinetics via two-color super-resolution microscopy. Nano Lett. 14, 339–345 10.1021/nl4041093 24303845 PMC3901574

[27] Ren P. H., Lauckner J. E., Kachirskaia I., Heuser J. E., Melki R., and Kopito R. R. (2009) Cytoplasmic penetration and persistent infection of mammalian cells by polyglutamine aggregates. Nat. Cell Biol. 11, 219–225 10.1038/ncb1830 19151706 PMC2757079

[28] Jullien R. (1987) Aggregation phenomena and fractal aggregates. Cont. Phys. 28, 477–493 10.1080/00107518708213736

[29] Keppler A., Gendreizig S., Gronemeyer T., Pick H., Vogel H., and Johnsson K. (2003) A general method for the covalent labeling of fusion proteins with small molecules *in vivo*. Nat. Biotechnol. 21, 86–89 10.1038/nbt765 12469133

[30] Mangiarini L., Sathasivam K., Seller M., Cozens B., Harper A., Hetherington C., Lawton M., Trottier Y., Lehrach H., Davies S. W., and Bates G. P. (1996) Exon I of the HD gene with an expanded CAG repeat is sufficient to cause a progressive neurological phenotype in transgenic mice. Cell 87, 493–506 10.1016/S0092-8674(00)81369-0 8898202

[31] Gandy S., and Heppner F. L. (2005) Breaking up (amyloid) is hard to do. PLoS Med. 2, 1228–1229 10.1371/journal.pmed.0020417 16363913 PMC1322301

[32] Waelter S., Boeddrich A., Lurz R., Scherzinger E., Lueder G., Lehrach H., and Wanker E. E. (2001) Accumulation of mutant huntingtin fragments in aggresome-like inclusion bodies as a result of insufficient protein degradation. Mol. Biol. Cell 12, 1393–1407 10.1091/mbc.12.5.1393 11359930 PMC34592

[33] Li X., Wang C. E., Huang S., Xu X., Li X. J., Li H., and Li S. (2010) Inhibiting the ubiquitin-proteasome system leads to preferential accumulation of toxic N-terminal mutant huntingtin fragments. Hum. Mol. Genet. 19, 2445–2455 10.1093/hmg/ddq127 20354076 PMC2876889

[34] Juenemann K., Schipper-Krom S., Wiemhoefer A., Kloss A., Sanz A. S., and Reits E. A. (2013) Expanded polyglutamine-containing N-terminal huntingtin fragments are entirely degraded by mammalian proteasomes. J. Biol. Chem. 288, 27068–27084 10.1074/jbc.M113.486076 23908352 PMC3779707

[35] Hoffner G., and Djian P. (2014) Monomeric, oligomeric and polymeric proteins in huntington disease and other diseases of polyglutamine expansion. Brain Sci. 4, 91–122 10.3390/brainsci4010091 24961702 PMC4066239

[36] Lajoie P., and Snapp E. L. (2010) Formation and toxicity of soluble polyglutamine oligomers in living cells. PLoS One 5, e15245 10.1371/journal.pone.0015245 21209946 PMC3011017

[37] Leitman J., Ulrich Hartl F., and Lederkremer G. Z. (2013) Soluble forms of polyQ-expanded huntingtin rather than large aggregates cause endoplasmic reticulum stress. Nat. Commun. 4, 2753 10.1038/ncomms3753 24217578

[38] Sathasivam K., Lane A., Legleiter J., Warley A., Woodman B., Finkbeiner S., Paganetti P., Muchowski P. J., Wilson S., and Bates G. P. (2010) Identical oligomeric and fibrillar structures captured from the brains of R6/2 and knock-in mouse models of Huntington's disease. Hum. Mol. Genet. 19, 65–78 10.1093/hmg/ddp467 19825844 PMC2792149

[39] Sathasivam K., Woodman B., Mahal A., Bertaux F., Wanker E. E., Shima D. T., and Bates G. P. (2001) Centrosome disorganization in fibroblast cultures derived from R6/2 Huntington's disease (HD) transgenic mice and HD patients. Hum. Mol. Genet. 10, 2425–2435 10.1093/hmg/10.21.2425 11689489

[40] Ströhl F., and Kaminski C. F. (2015) A joint Richardson–Lucy deconvolution algorithm for the reconstruction of multifocal structured illumination microscopy data. Methods Appl. Fluoresc. 3, 14002 10.1088/2050-6120/3/1/014002 29148478

