# Supplementary material for: Live-cell super-resolution microscopy reveals a primary role for diffusion in polyglutamine-driven aggresome assembly
Source: J Biol Chem. 2018 Nov 6;294(1):257–68. doi: 10.1074/jbc.RA118.003500 (PMC6322900; doi:10.1074/jbc.RA118.003500)
Supplement: Supporting Information [file supp_294_1_257__index.html]

Live-cell super-resolution microscopy reveals a primary role for diffusion in polyglutamine-driven aggresome assembly — Mechanisms of aggresome formation — Live-cell super-resolution microscopy reveals a primary role for diffusion in polyglutamine-driven aggresome assembly — Mechanisms of aggresome formation — Supporting Information 

# Live-cell super-resolution microscopy reveals a primary role for diffusion in polyglutamine-driven aggresome assembly

## Supporting Information

- Supporting Information - Figures and legends
- Live-cell super-resolution microscopy reveals a primary role for diffusion in polyglutamine aggresome assembly - video 1
- Live-cell super-resolution microscopy reveals a primary role for diffusion in polyglutamine aggresome assembly - video 2
- Live-cell super-resolution microscopy reveals a primary role for diffusion in polyglutamine aggresome assembly - video 3
- Live-cell super-resolution microscopy reveals a primary role for diffusion in polyglutamine aggresome assembly - video 4
- Live-cell super-resolution microscopy reveals a primary role for diffusion in polyglutamine aggresome assembly - video 5
