# Supplementary material for: Live-cell super-resolution microscopy reveals a primary role for diffusion in polyglutamine-driven aggresome assembly
Source: J Biol Chem. 2018 Nov 6;294(1):257–68. doi: 10.1074/jbc.RA118.003500 (PMC6322900; doi:10.1074/jbc.RA118.003500)
Supplement: Supporting Information [file supp_RA118.003500_137495_2_supp_226779_phdrj1.docx]

Live-cell super-resolution microscopy reveals a primary role for diffusion in polyglutamine-driven aggresome assembly

**Meng Lu^1^, Luca Banetta^2,1^, Laurence J. Young^1,^** ^†^**^, #^, Edward J. Smith^3^, Gillian P. Bates^3^, Alessio Zaccone^2^, Gabriele S. Kaminski^1^, Alan Tunnacliffe^1^, Clemens F. Kaminski^1,*^**

^1^Cambridge Infinitus Research Center, Department of Chemical Engineering and Biotechnology, University of Cambridge, Cambridge, CB3 0AS, United Kingdom

^2^Department of Chemical Engineering and Biotechnology, University of Cambridge, Cambridge, CB3 0AS, United Kingdom

^3^Sobell Department of Motor Neuroscience and Movement Disorders and Huntington’s Disease Center, Institute of Neurology, University College London, London, WC1N 3BG, United Kingdom

**Supporting Information**

**Figure S1**

**(A)** Confocal images of an EGFP-HDQ72 cell with an aggresome visible in the perinuclear area.

**(B**) Proportion of EGFP-HDQ72 cells containing aggregates at different time points after tetracycline induction. Three independent experiments were performed with around 200 cells assessed per population per experiment.

**Figure S2**

Quantification of aggregate in long distance motion (>2 μm). 35537 aggregates from normal cells and 7410 aggregates from nocodazole treated cells were analysed.

**Figure S3**

Quantification of aggregate cluster numbers of different length scales. Cell samples were fixed and 279 aggregate clusters were recorded and analysed by 3D SIM.

**Figure S4**

Examples of binary images of aggregates used for gyration radius analysis. Scale bar: 1 μm.

**Video 1**. High-speed SIM recording (2 Hz) of cytosolic polyQ aggregates.

**Video 2**. High-speed SIM recording (6 Hz) of aggregate particles in quasi-random motion.

**Video 3**. High-speed SIM recording (2 Hz) of small cluster motion within an aggresome.

**Video 4**. 3D SIM reconstruction of an aggresome and small aggregate clusters.

**Video 5**. 3D SIM reconstruction of a small, amorphous cluster.

# **Mathematical model of aggresome growth**

To support and better understand the experimental observations, a mathematical model wasdeveloped to describe the formation and growth of the polyQ perinuclear aggresome.

The system was modelled to be composed of a spherical aggresome, considered to be a fractal aggregate with well-defined radius of gyration and made of elementary building blocks (clusters), all with the same average size and effective diffusion coefficient. The aggresome is located at the centre of a cell modelled as a sphere. The cytosol is populated homogeneously by clusters taken to be spherical objects of identical size; the presence of monomeric polyQ is neglected.

A spherical symmetry was assumed for the problem, so that the concentration of polyQ in the liquid phase, expressed as *clusters/m*^3^, depends on time and radial coordinates only. The model describes the growth of a single large aggresome at the center of the cell, neglecting the possibility of multiple aggresomes forming simultaneously.

Transport of the clusters from the cytosol to the aggresome was modelled to be caused by a combination of Brownian motion along the radial direction and active transport through via MTs. The two contributions were considered to be independent from another, so when a cluster enters the MTOC region it is no longer affected by diffusive transport, which only takes place inside the cytosol. The initial concentration of clusters inside the cytolsol was estimated from analysis of the microscopy data. The analysis revealed that only 5% of the total clusters are under active transport at any time and this information was used to initialise the input parameters for the model.

The model reduces to a moving-boundary Stefan problem where two distinct transport processes take place at the same time. This leads to two separate Cauchy problems, for diffusive and active transport, respectively, in a spherical geometry with a moving-boundary given by the interface between solid aggresome (growing as a function of time) and the liquid-like cytosol, which fills the surrounding space. Conservation of matter at the solid-liquid - (or aggresome-liquid -) interface is implemented to describe the quantity of polyQ migrating into the growing aggresome and to update the position of the radius of the increasing aggresome (the moving lower boundary of the domain where the differential equations are solved).

The two Cauchy problems were formulated for diffusive and active transport as per equations (1)-(7). Every building-block (cluster) is either moving under Brownian motion in the cytosol or being actively transport (advected) inside one of the MTOC. The equations for the diffusive transport with boundary and initial conditions are:


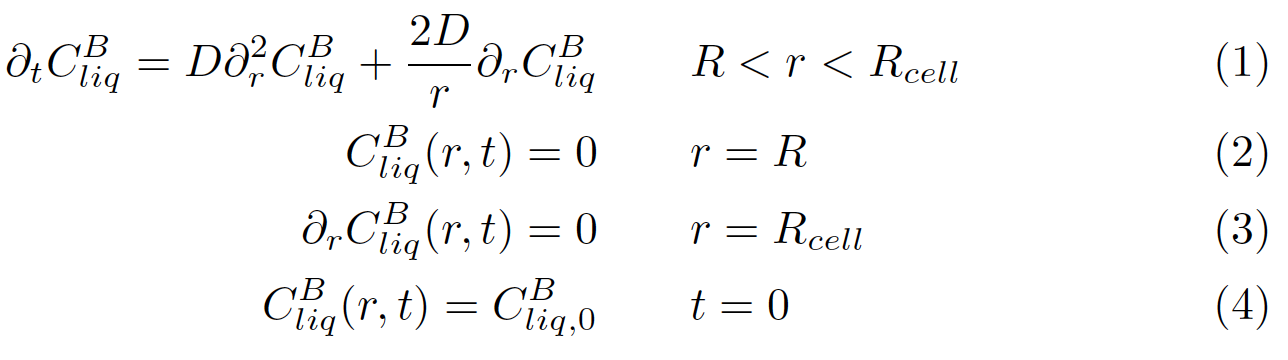


while for the advective transport in the MTOC the following pure-advection equation was used with approproateboundary conditions:

*
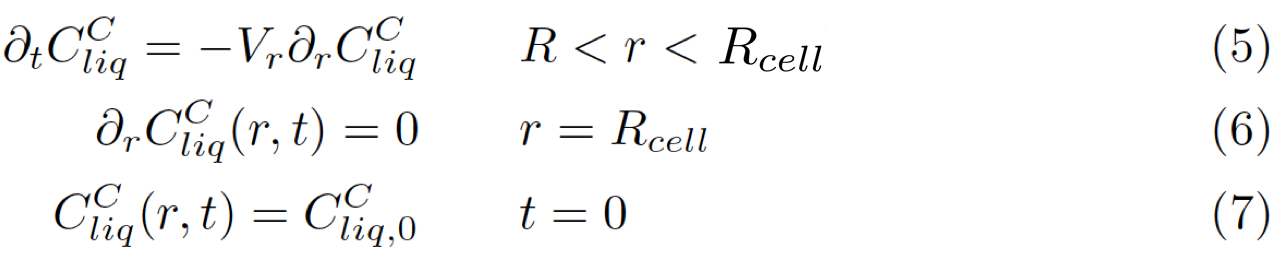
*

*­*

*D* is the diffusion coefficient, considered to be constant along the radial coordinate, *V_r_* is the radial component of the advection speed inside the MTOC (taken to be independent of *r*, based on experimental evidence), *R* the radius of the growing aggresome corresponding to the position of the solid-liquid interface during time, *R_cell_* the characteristic size of the cell,
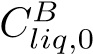
 the initial concentration of clusters inside the cytosol that are transported by Brownian motion, and
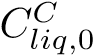
 is the initial concentration of clusters inside the MTOC that are subsequently transported by pure advection to the aggresome.

The update of the solid-liquid interface is obtained by enforcing conservation of matter at the aggresome interface *r* = *R*: the RHS of equation (8) below represents the flux of matter that is included in the solid phase during the time span *δt*. This leads to the deposition of an external shell of aggregates to the surface of the aggresome that causes an increase of the mass of the aggresome expressed in the LHS of (8).


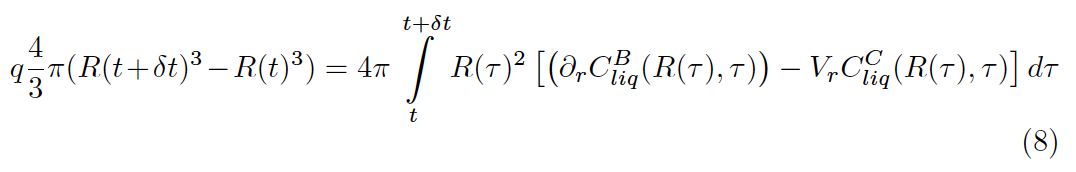


In this case *τ* represents the temporal variable integrated between *t* and *t* + *δt*, meanwhile *q* represents the amount of matter necessary to increase the system by a unit of volume. Because the aggresome is a fractal aggregate, its density (number of building blocks per unit volume) is not constant, and decreases over time because of the spatially self-similar nature of the growth process. It is possible to relate *q*(*t*) to the fractal dimension *d_f_* of the aggresome and its radius of gyration *R*, and to the radius of the single building block (cluster) *a* via the following relation


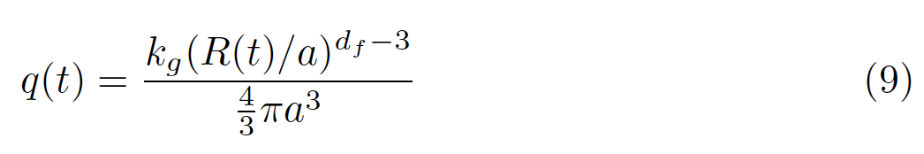


*
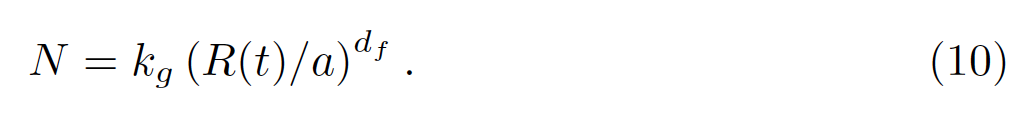
*The fractal scaling prefactor *k_g_* and the fractal dimension *d_f_* are related to the number of building blocks *N* inside the aggresome via the standard fractal scaling relation

Even though *q* varies with time overall, it will be considered constant over the small time-step *δt* taken for the discretization of the transport equations. Dividing both intervals by *δt* and doing the limit for *δt* → 0 we obtain


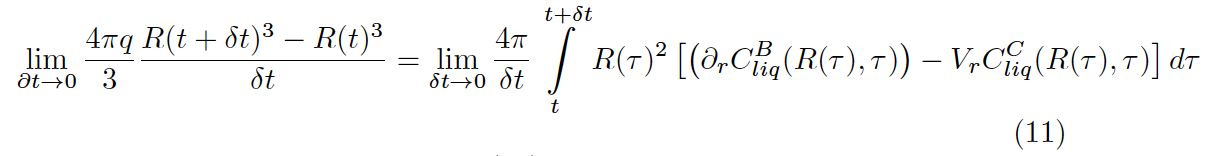


Because the integrand function in (11) is continuous and limited inside every interval [*t,t*+*δt*] it is possible to apply the Mean Value Theorem (MVT) defined in (12)


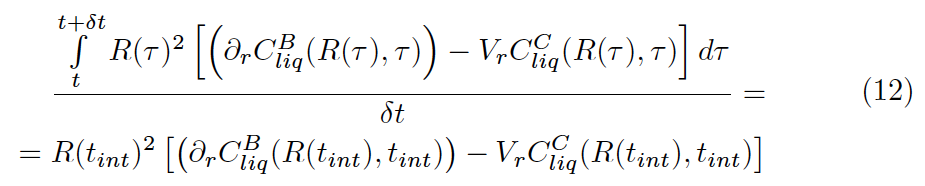


where *t_int_* is an intermediate point inside the interval [*t,t* + *δt*]. Applying MVT together with the definition of the first derivative IN (11) we obtain


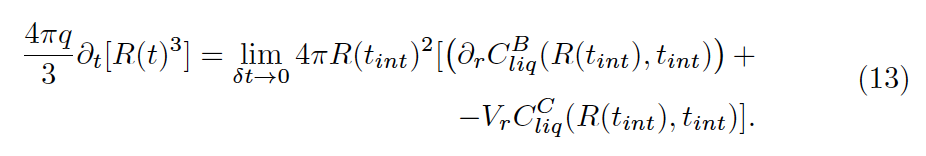


*
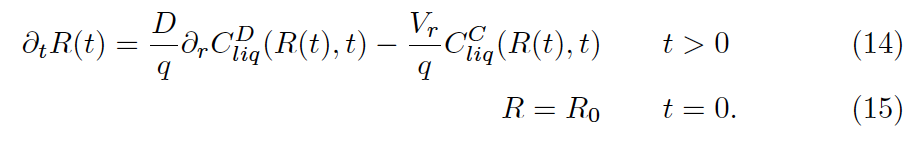
*Finally (13) can be reduced to the following final equation

Equation (14) is numerically solved with an explicit first order finite difference scheme where *q* stays constant over each time span and equal to its value related to the lower limit of every interval; time step were chosen such that *q* was approximately constant over each time interval.

# **2. Numerical solution**

The system (1)-(4) can be numerically solved by the Crank-Nicholson method: the continuous space coordinate is divided into a series of *N* discrete intervals and the implicit finite difference scheme is applied for *j* = *n*+1*,..,N* where *n*+1 is the node related to the solid-liquid interface; (16) is a general line of the linear system that needs to be solved.


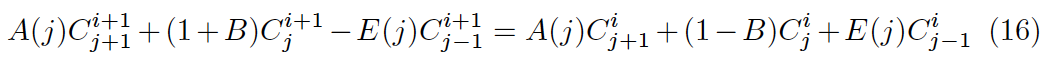


The values of the coefficients A(j), B and E(j) are reported in (17) - (19)


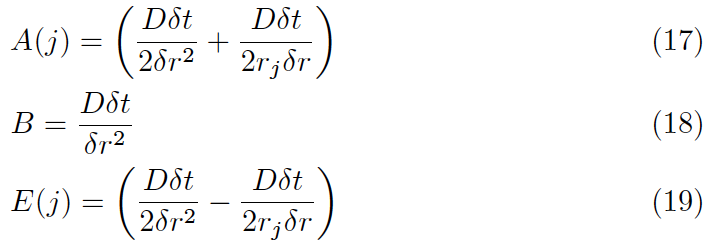


*δr* is the interval between two consecutive nodes and *δt* is the time step adopted in this work.

The two boundary conditions (2)-(3) can be respectively written as


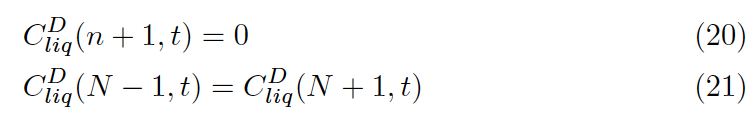


The second represents a symmetry condition that, thanks to the use of a ghost node *N* +1: the *N^th^* point of the grid can be used as a mirror between neighbouring cells so that coefficients of the last line of the linear system become


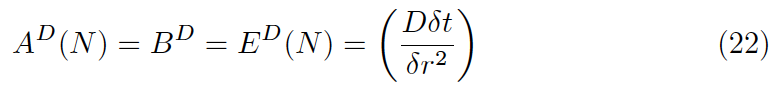


Regarding the other Cauchy problem, it has been demonstrated that the analytical solution of a pure convection equation, knowing the initial concentration


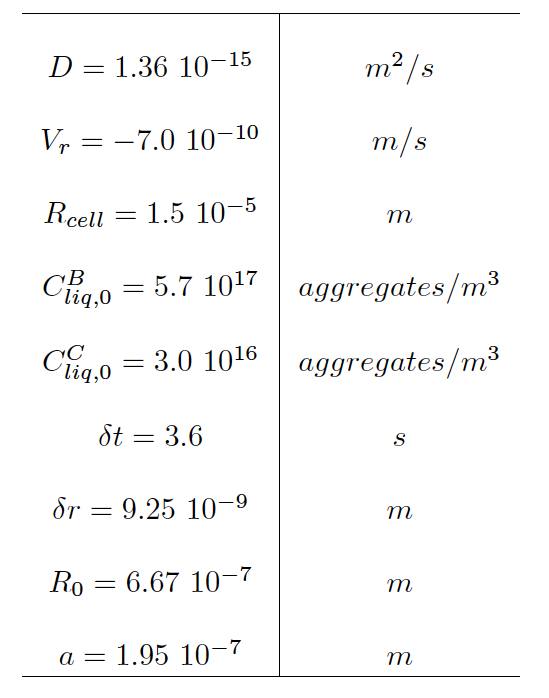


Table 1: list of input data together with the respective SI units adopted in this work distribution


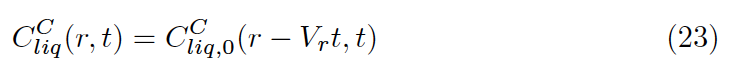
*C_liq,_^C^* _0_, is

In the main text we describe the theoretical approach to update the position of the solid-liquid interface, what follows is the explicit first order discretization adopted in this work


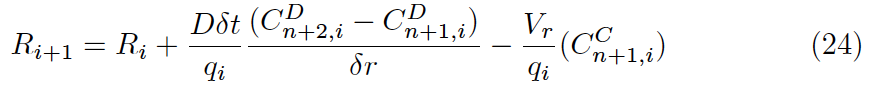


The evaluation of *q_i_* at each time step can be done through equation (9), subsequent to evaluation of *k_g_* and *d_f_*  via (10). Using experimental data these were determined to be *k_g_* = 1*.*75 and *d_f_* = 2*.*08. The complete list of input data is shown in Table 1; the value of *V_r_* is negative because the direction of the active transport is opposite to the radial direction as defined here.
